# Comparison of Three Algorithms for the Retrieval of Land Surface Temperature from Landsat 8 Images

**DOI:** 10.3390/s19225049

**Published:** 2019-11-19

**Authors:** Lei Wang, Yao Lu, Yunlong Yao

**Affiliations:** 1 College of Wildlife Resources, Northeast Forestry University, Harbin 150040, China; wanglei_happiness@163.com; 2 Key Lab for Garden Plant Germplasm Development & Landscape Eco-restoration in Cold Regions of Heilongjiang Province, Harbin 150040, China; 3 College of Architectural Engineering, Heilongjiang University of Science and Technology, Harbin 150022, China; luyao19930307@gmail.com

**Keywords:** Landsat 8 TIR data, land surface temperature, mono-window algorithm, split window algorithm, single-channel method, sensitivity analysis

## Abstract

The successful launch of the Landsat 8 satellite provides important data for the monitoring of urban heat island effects. Since the Landsat 8 TIRS data has two thermal infrared bands, it is suitable for many algorithms to retrieve the land surface temperature (LST). However, the selection of algorithms for retrieving the LST, the acquisition of algorithm input parameters, and the verification of the results are problems without obvious solutions. Taking Changchun City as an example, this paper used the mono-window algorithm (MWA), the split window algorithm (SWA), and the single-channel (SC) method to extract the LST from the Landsat 8 image and compared the three algorithms in terms of input parameters, accuracy, and sensitivity. The results show that all three algorithms can achieve good results in retrieving the LST. The SWA is the least sensitive to the error of the input parameters. The MWA and the SC method are sensitive to the error of the input parameters, and compared with the error of the LSE, these two algorithms are more sensitive to the error of atmospheric water vapor content. In addition, the MWA is also very sensitive to the error of the effective mean atmospheric temperature.

## 1. Introduction

Land surface temperature (LST) is the direct driving factor for water heat exchange between the Earth’s surface and the atmosphere, and is the key parameter in many physical processes [1,2,3]. Retrieving LST from thermal infrared remote sensing data at global, regional, and urban scales have unparalleled advantages, and this is the most common method for studying urban heat island effects. Since its launch on 11 February 2013, the Landsat 8 satellite has ingested and transmitted over 500 multispectral-image scenes to the ground every day; the revisit time of Landsat 8 is 16 days and together with Landsat 7 ETM+ constitutes an 8 day interval Landsat repeat observation cycle [4]. It provides extremely valuable data for the observation of surface temperatures. The Landsat 8 carries two main sensors: Operational Land Imager (OLI) and Thermal Infrared Sensor (TIRS). The TIRS contains two thermal infrared bans, band 10 and band 11, with a resolution of 100 m which enable the split window algorithm to be used on Landsat 8. But shortly after launching, it was observed for the Landsat 8-TIRS (L8-TIRS) bands that radiance from outside of the instrument’s field-of-view produced a non-uniform ghost signal across the focal plane that varied depending on the out-of-scene content. This stray light effect was approximately 8% or higher in the emittance received in band 11, which was twice than that of band 10 [5,6]. Because of the large calibration uncertainty, the United States Geological Survey (USGS) does not recommend the use of band 11 for the split window algorithm [7]. In 2015, Gerace et al. [6] proposed a correction algorithm named the stray light correction algorithm (SLCA) to reduce the error caused by stray light which greatly reduces the calibration error caused by stray light. In February 2017, the USGS started to implement the SLCA to Landsat 8 data; since then, it has been applied to all new and prior Landsat 8 acquisitions. Nevertheless, the Landsat team still does not recommend the use of band 11 for the split window algorithm, because, whether this correction is accurate, it requires more research to prove. Although the SLCA has been applied to Landsat 8 for over two years, many scholars have verified the accuracy of the 11th band [8,9]. But more research is needed to prove that the split window algorithm can also be used for Landsat 8.

Retrieving LST from the thermal infrared band of remote sensing has been a difficult problem. Many scholars have studied the method for LST retrieval; among these methods, there are three methods that are widely used: the mono-window algorithm (MWA), developed by Qin et al. [10], the split window algorithm (SWA), developed by Mcmillin et al. [11], and the single-channel (SC) method, developed by Jiménez-Muñoz and Sobrino et al. [12]. These three algorithms were originally proposed for other thermal infrared remote sensing; when applied to Landsat 8 TIR data, they should be improved. Wang et al. [13] proposed an improved mono-window (IMW) algorithm based on Qin’s MWA, which makes the MWA appropriate for Landsat 8. The average bias and root mean square error (RMSE) of the estimated LST derived by the IMW algorithm are −0.05 K and 0.84 K, respectively. Rozenstein et al. [14] improved the SWA and derived LST from Landsat 8 TIR data. The RMSE of the simulated LST was calculated as 0.93 °C. For the SC method, Jiménez-Muñoz and Sobrino et al. [15] made an improvement for Landsat 8 in 2014 and verified the accuracy; the results showed that, when the water vapor content is below 3g/cm^2^, the RMSE is less than 1.5 K. In addition, there are other scholars who have improved these algorithms and have received good results [16,17]. When these algorithms are applied for LST retrieval, some corresponding input parameters are needed; land surface emissivity (LSE) and atmospheric transmittance (τ) are essential for all of these three methods. In addition, the MWA needs one more parameter, the effective mean atmospheric temperature ($T_{a}$). In the acquisition of these parameters, scholars use many different methods [18,19,20,21]. Taking the acquisition of atmospheric transmittance as an example, some scholars calculate the atmospheric transmittance from water vapor content [10], some obtain it from the USGS website based on the time of image acquisition and the central latitude and longitude of image [22]. The diversity of algorithms and the variety of input parameter acquisition methods make it difficult for scholars to choose the proper method when retrieving LST.

In addition, after the LST is retrieved, it is difficult to verify the results. There are many studies exploring how to verify the retrieved LST. In general, these verification methods can be divided into three types: temperature-based method (directly compares the satellite-derived LST with in situ LST measurements at the satellite overpass) [16,23,24]; radiance-based method [10,25,26]; and cross validation method (validates the derived LST with other LST product) [27,28,29]. Whether the retrieved LST can be used in further research depends on the accuracy of the results, so it is vital to choose an effective verification method.

In summary, there are three major problems in retrieving surface temperature using Landsat 8 remote sensing imagery. (1) How to choose the appropriate algorithm to retrieve the surface temperature from Landsat 8 image? (2) How to obtain the input parameters of each algorithm? (3) How to verify the retrieved LST? In order to solve these three problems, this paper takes Changchun City, Jilin Province, China, as an example and extracts the LST from the Landsat 8 image by the improved mono-window algorithm (MWA), split window algorithm (SWA), and single-channel (SC) method. Detailed explanations of the calculation method and operation flow of the three algorithms in addition to the input parameters of each algorithm and its obtention method is given too. Among these input parameters, considering the existence of many red and blue roofs in Changchun (the NDVI (normalized difference vegetation index) values of these pixels are abnormal and are easily misclassified, which may lead to error in the retrieved LST), we have improved the NDVI threshold method to make the calculated surface emissivity (LSE) more accurate. For the retrieved LST verification, we compared the advantages and limitations of three different temperature verification methods in detail and then proposed a verification methods according to the data we had obtained, that is, verifying the retrieved LST with the air temperature recorded by the weather station in the area with high vegetation coverage. Finally, the sensitivity analysis of the three algorithms is carried out. Through the sensitivity analysis, the applicable conditions of each algorithm can be learned. Our findings will help people to choose the appropriate algorithm for LST retrieval. At the same time, this paper uses the split window algorithm to retrieve LST and verify its accuracy, which is also meaningful to judge whether the corrected Landsat 8 TIRS data are accurate.

## 2. Materials and Methods 

### 2.1. Study Area

**Figure 1:**
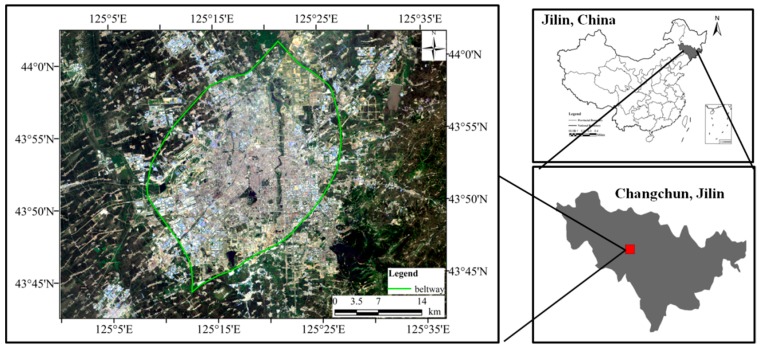
The location of Changchun City.

Changchun City (125°06’–125°36’ E, 43°43’~44°04’ N) is the capital of Jilin Province, China, the central city, the industrial base, and comprehensive transportation hub of China (Figure 1). The superior geographical position and the important core functions of region’s economy and culture made Changchun develop rapidly. In the past 30 years, Changchun experienced rapid urbanization, and the urban built-up area expanded rapidly, from 143.15 km^2^ in 1984 to 577.45 km^2^ in 2014 [16]. In the process of rapid urbanization, the living standards of citizens in Changchun City have improved, but at the same time, they have also been facing many environmental problems such as the urban heat island effect.

According to meteorological statistics from Changchun City, the average annual temperature in Changchun increased by 1.86 °C during the 60 years from 1951 to 2011 [30]. Studies have shown that due to the fact of urbanization, the average temperature of Changchun’s cities is 0.1–0.5 °C higher than that of surrounding rural areas [16], and the proportion of Changchun’s heat island area has increased from 15.27% in 1984 to 29.62% in 2014 [22]. These studies have shown that the urban thermal environment in Changchun is deteriorating. Studying the spatial differentiation of Changchun’s LST will not only help people grasp the thermal environment of Changchun City but will also be helpful for further study of the optimization of urban green spaces. It is also of great importance for mitigating the urban heat island effect and improving the urban thermal environment which is conducive to people’s physical and mental health and reducing urban energy consumption.

### 2.2. Data Preprocessing

The images used in this article were Landsat 8 images of the Changchun area acquired on 4 July 2016, downloaded from the USGS and which were randomly selected from images of good quality in the summer of the study area. The images were Level 1T terrain correction images with the projection coordinate system of UTM-WGS84, and they were orthorectified using ground control points and digital elevation model (DEM) data. Thus, no geometric correction was needed when processing the Landsat 8 images. Preprocessing of the Landsat 8 images included image cropping, radiometric calibration, and atmospheric correction. For Landsat 8 OLI data, radiometric calibration was required to convert the digital number (DN) value to emissivity, and then the atmospheric correction was performed to eliminate the influence of atmospheric and illumination factors on the radiation value received by the sensor so that the NDVI could be calculated using the corrected image. For Landsat 8 TIR data, radiometric calibration was performed to convert the DN values of Band 10 and Band 11 to brightness temperature. According to the USGS announcement, the DN value of the Landsat 8 TIR data can be converted into thermal spectral radiance by Formula (1), and then the spectral radiance can be converted to brightness temperature by the Planck radiation function seen in Formula (2).

(1) $$R_{i}=M_{i}Q_{i}+A_{i}-O_{i}$$

(2) $$T_{i}=\frac{K_{2}}{\ln\left(1+K_{1}/R_{i}\right)}$$

where $R_{i}$ is the spectral radiance ($W\cdot m^{-2}\cdot {\mathrm{sr}}^{-1}\cdot {\mu m}^{-1}$) of band I; $Q_{i}$ is the DN value of band I; $T_{i}$ is the brightness temperature of ban I, and $M_{i}\;$, $A_{i}$, $O_{i}$, $K_{2}$, and $K_{1}$ are constants, which can be searched from the MTL file of Landsat 8; their values are shown in Table 1. It should be noted that since 3 February 2014, $O_{i}$ should not be considered.

### 2.3. Algorithms and Parameter Calculation

#### 2.3.1. Mono-Window Algorithm (MWA)

Qin’s mono-window algorithm is shown in Formula (3). This algorithm requires three parameters: the effective mean atmospheric temperature (T_a_), land surface emissivity (ε), and atmospheric transmittance (τ). However, Formula (3) is proposed for TM images. The images used in this study were Landsat 8 images, so this method needs to be improved to make it suitable for new data. Fei Wang et al. [13] improved the algorithm and made it suitable for Band 10 of Landsat 8. This improved algorithm is called improved mono-window algorithm as is shown in Formula (4).

T_s_ = [a_6_(1 − C_6_ − D_6_)+(b_6_(1 − C_6_ − D_6_) + C_6_ + D_6_) · T_6_ − D_6_T_a_]/C_6_ (3)

T_s_ = [a_10_(1 − C_10_ − D_10_) + (b_10_(1 − C_10_ − D_10_) + C_10_ + D_10_) · T_10_ − D_10_T_a_]/C_10_ (4)

where a_10_ and b_10_ are constants, and their values are different in different temperature ranges as is shown in Table 2. Both C_10_ and D_10_ are functions of LSE (ε_10_) and atmospheric transmittance (τ_10_), and the calculation methods are as shown in Equations (5) and (6), respectively. T_a_ is the effective mean atmospheric temperature.

C_10_ = ε_10_ × τ_10_ (5)

D_10_ = (1 − τ_10_) × [1 + (1 − ε_10_) × τ_10_] (6)

Land surface emissivity (ε_10_) is a crucial parameter in retrieving LST. Different emissive materials have different emissivity due to the fact of their different materials, roughness, and even observation angles. To calculate the LSE, the classification-based emissivity method [29,31] and NDVI-based emissivity method [32] are commonly used. Among them, the NDVI-based emissivity method proposed by José A et al. [21] is more widely used, because this method does not consider the influence of surface roughness and has higher accuracy at 10–12 μm. The principle of the NDVI threshold method is to divide the pixels of an image into three categories based on the NDVI value with a certain threshold, pure vegetation pixel, bare land pixel, and mixed pixel, and then to calculate the surface emissivity according to Formula (7), in which P_v_ is the proportion of vegetation and can be calculated by Formula (8), and NDVI can be calculated by Formula (9).

(7) $$\varepsilon_{\lambda }=\{\begin{array}{cc} \varepsilon_{s\lambda }, & \mathrm{NDVI}<{\mathrm{NDVI}}_{s}\\ \varepsilon_{s\lambda }+(\varepsilon_{v\lambda }-\varepsilon_{s\lambda })P_{v}, & {\mathrm{NDVI}}_{s}\leq \mathrm{NDVI}\leq {\mathrm{NDVI}}_{v}\\ \varepsilon_{v\lambda }, & \mathrm{NDVI}>{\mathrm{NDVI}}_{v} \end{array}$$

(8) $$P_{v}={\left[\frac{\mathrm{NDVI}-{\mathrm{NDVI}}_{s}}{{\mathrm{NDVI}}_{v}-{\mathrm{NDVI}}_{s}}\right]}^{2}$$

(9) $$\mathrm{NDVI}=\frac{\mathrm{NIR}-R}{\mathrm{NIR}+R}$$

where $\varepsilon_{\lambda }$ is LSE; $\varepsilon_{v\lambda }$ and $\varepsilon_{s\lambda }$ are, respectively, the vegetation and soil emissivity; ${\mathrm{NDVI}}_{s}$ is the NDVI of bare land pixels (the value usually assigned is 0.2); similarly, ${\mathrm{NDVI}}_{v}$ is the NDVI of pure vegetation pixels (the value assigned was 0.5); NIR is the near infrared ban (5 for Landsat 8), and R is the red band (band 4 for Landsat 8). When NDVI ≤ 0, the pixel can be regarded as water, the LSE value is 0.991 for band 10 and 0.986 for band 11; when 0 < $\mathrm{NDVI}<{\mathrm{NDVI}}_{s}$, the pixel can be regarded as bare land, the value is 0.964 and 0.970 for band 10 and band 11, respectively. When $\mathrm{NDVI}>{\mathrm{NDVI}}_{v}$, the pixel can be regarded as pure vegetation, the value is 0.984 and 0.980 for band 10 and band 11, respectively [17]. When ${\mathrm{NDVI}}_{s}\leq \;\mathrm{NDVI}\leq {\;\mathrm{NDVI}}_{v}$, these areas are considered to be mixed pixels, the LSE value can be calculated by Formula (7). In addition, it should be noted that in recent years, in urban areas, there are many roofs made of electro-galvanized steel, generally blue. These materials generally have a NDVI value higher than 0.5 and are easily classified into pure vegetation pixels. The NDVI value of the red roof is generally lower than 0, which is easily erroneously classified into water pixels. This classification error can lead to the error in retrieved LST. Therefore, it is necessary to separate the two types of pixels in advance and separately assign the emissivity value. The average LSE value of these typical materials is shown in Table 3.

For the effective mean atmospheric temperature (T_a_), Qin et al. [10] gives the linear relationship between the effective mean atmospheric temperature (Ta) and the near-surface air temperature ($T_{0}$) at different conditions, as shown in Table 4. Atmospheric transmittance is the last parameter for the MWA. There are several ways to obtain the atmospheric transmittance. For example, Yang et al. [22] obtain it from the USGS website based on the time of image acquisition and the central latitude and longitude of image. Ahn et al. [18] used the simplified Planck formula to directly calculate the atmospheric transmittance without considering the atmospheric effect. Barsi et al. [33] uses NASA’s atmospheric correction parameter calculator to estimate atmospheric transmittance. Since atmospheric transmittance is mainly affected by air humidity, using MODTRAN to simulate the linear relationship between atmospheric transmittance and atmospheric water vapor content (ω) is the most used method [14,17,29,34]. In this paper, the results of the Rozenstein’s [14] simulation were used to calculate the atmospheric transmittance. The atmosphere absorbs light of different wavelengths differently. The linear relationship between the atmospheric transmittance (τ) and the atmospheric water vapor content ω on the 10th and 11th bands of Landsat 8 is shown in Table 5.

**Figure 2:**
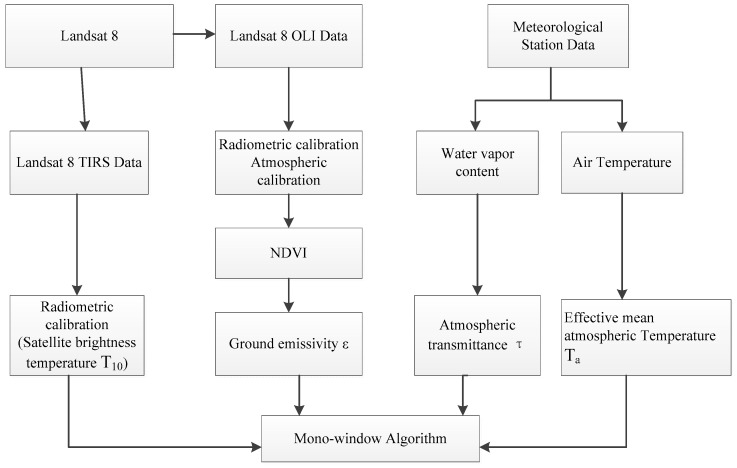
Flow chart of the mono-window algorithm.

Through the above description, we can summarize the operation flow of the MWA as Figure 2:

#### 2.3.2. Split Window Algorithm (SWA)

The split window algorithm (SWA) was originally proposed by McMillin et al. [11]. It is an algorithm for observing the ocean surface temperature based on AVHRR thermal infrared data. The principle is that two adjacent thermal infrared bands have different absorption characteristics; the attenuation information of the atmosphere on the thermal radiation can be obtained by the difference between the brightness temperatures of the two TIR bands. Price et al. [35] used the SWA to retrieve the LST. The SWA is widely used due to the fact that this algorithm has less dependence on atmospheric parameters and is simple to operate. At present, there are many studies on the SWA [14,29,36]. Rozenstein et al. [14] have improved the SWA for the Landsat 8 image, and simplified the formula to make it clean; thus, his method was selected in this paper.

Compared with the MWA, the SWA only needs two parameters: atmospheric transmittance (τ) and LSE (ε). The calculation formula for the SWA is as follows:

(10) $$T_{s}=A_{0}+A_{1}T_{10}-A_{2}T_{11}$$

where $T_{s}$ is the LST and $T_{10}$ and $T_{11}$ are the brightness temperature of band 10 and band 11, respectively. $A_{0}$, $A_{1}$, and $A_{2}$ are the parameters and can be calculated through the following formulas.

(11) $$A_{0}=E_{1}a_{10}+E_{2}a_{11}$$

(12) $$A_{1}=1+A+E_{1}b_{10}$$

(13) $$A_{2}=A+E_{2}b_{11}$$

(14) $$C_{i}=\varepsilon_{i}\tau_{i}$$

(15) $$D_{i}=\left(1-\tau_{i}\right)\left[1+\left(1-\varepsilon_{i}\right)\tau_{i}\right]$$

(16) $$A=D_{10}/E_{0}$$

(17) $$E_{1}=D_{11}\left(1-C_{10}-D_{10}\right)/E_{0}$$

(18) $$E_{2}=D_{10}\left(1-C_{11}-D_{11}\right)/E_{0}$$

(19) $$E_{0}=D_{11}C_{10}-D_{10}C_{11}$$

where εi is the LSE of band I; τi is the atmospheric transmittance of band i; the ways to calculate them are given above. The difference is that for the SWA, the LSE and atmospheric transmittance should be calculated separately for band 10 and band 11. $a_{10}$, $b_{10}$, $a_{11}$, and $b_{11}$ are constants; their values are given by Rozenstein et al. [14] as is shown in Table 6.

**Figure 3:**
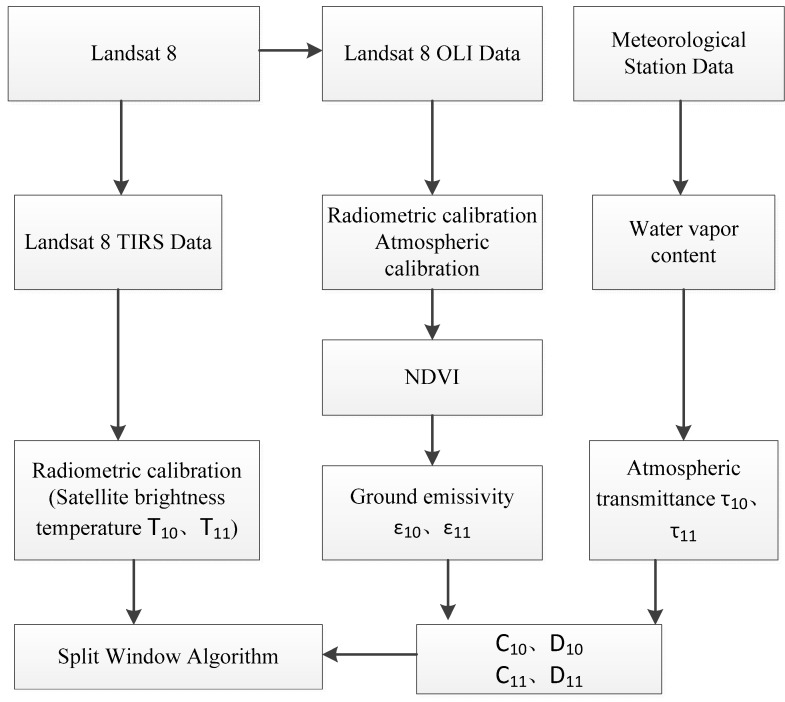
Flow chart of the split window algorithm.

Through the above description, we can summarize the operation flow of the SWA as Figure 3:

#### 2.3.3. Single-Channel (SC) Method

The single-channel (SC) method was proposed in 2003 [12] and improved in 2009 [37] by Jiménez-Muñoz and Sobrino. This algorithm requires only two input parameters, the LSE (ε) and the atmospheric water vapor content (ω). Differing from the MWA, the SC method does not need the effective mean atmospheric temperature ($T_{a}$) parameter, and the atmospheric water vapor content is not required to be calculated to the atmospheric transmittance which reduces the error of the final retrieved LST due to the error of the effective mean atmospheric temperature ($T_{a}$). For these advantages, after the single-channel algorithm was proposed, it was used by many scholars for various types of thermal infrared remote sensing, such as Landsat 5 TM, Landsat 7 ETM+, MODIS, ASTER, and ENVISAT AATSR [19,20,37,38]. In 2014, Jiménez-Muñoz and Sobrino [15] improved the SC method for Landsat 8 and calculated the corresponding parameters, the formulas of the SC method are shown as follow:

(20) $$T_{s}=\gamma \left[\frac{1}{\varepsilon }\left(\Psi_{1}L_{\mathrm{sen}}+\Psi_{2}\right)+\Psi_{3}\right]+\delta$$

where $T_{s}$ is the LST; ε is LSE; γ and δ are two parameters depending on the Planck function and can be calculated by Formulas (21) and (22); in the formulas, $L_{\mathrm{sen}}$ is at-sensor registered radiance (W/(m^2^·sr·μm)); $T_{\mathrm{sen}}$ is the at-sensor brigntness temperature; for band 10 of Landsat 8, $b_{\gamma }$ = 1324.

Atmospheric function parameters $\Psi_{1}$, $\Psi_{2},$ and $\Psi_{3}$ can be calculated through Formula (23).

(21) $$\gamma \approx \frac{T_{\mathrm{sen}}^{2}}{b_{\gamma }L_{\mathrm{sen}}}$$

(22) $$\delta \approx T_{\mathrm{sen}}-\frac{T_{\mathrm{sen}}^{2}}{b_{\gamma }}$$

(23) $$\left[\begin{array}{c} \Psi_{1}\\ \Psi_{2}\\ \Psi_{3} \end{array}\right]=\left[\begin{array}{ccc} c_{11} & c_{12} & c_{13}\\ c_{21} & c_{22} & c_{23}\\ c_{31} & c_{32} & c_{33} \end{array}\right]\left[\begin{array}{c} \omega^{2}\\ \omega \\ 1 \end{array}\right]$$

For band 10 of Landsat 8:

(24) $$C=\left[\begin{array}{ccc} 0.04019 & 0.02916 & 1.01523\\ -0.38333 & -1.50294 & 0.20324\\ 0.00918 & 1.36072 & -0.27514 \end{array}\right]$$

**Figure 4:**
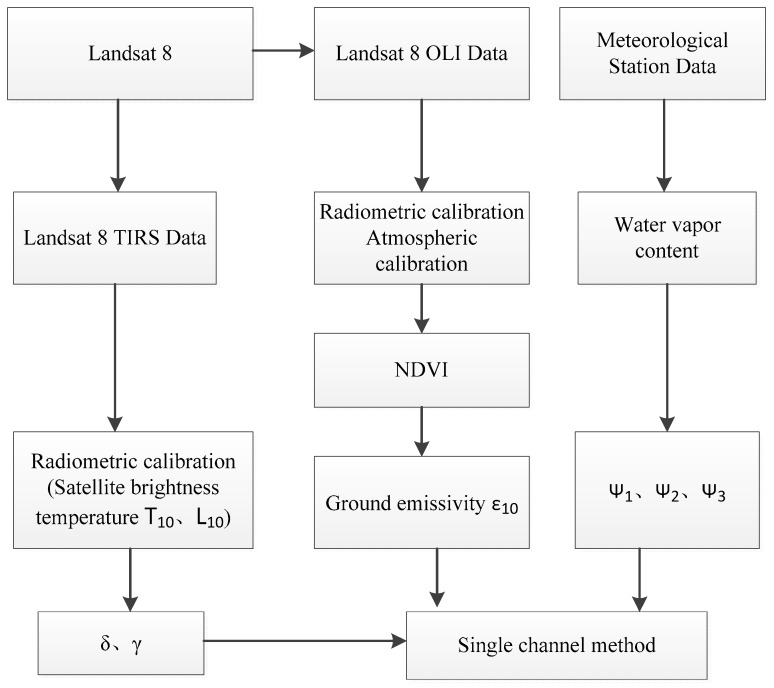
Flow chart of the single-channel method.

Through the above description, we can summarize the operation flow of the SC method as Figure 4:

### 2.4. Sensitivity Analysis of the Three Algorithms

Due to the difficulties in acquiring the parameters of each algorithm, these input parameters will inevitably have errors which will further lead to errors in the results. Thus, it is necessary to evaluate the influence of the error of the input parameters on the results. Therefore, a sensitivity analysis of each algorithm is required. We used Equation (25) to calculate the effect of the error of the input parameters on the results:

(25) $${\Delta T}_{s}=\left|T_{s}\left(x+\Delta x\right)-T_{s}\left(x\right)\right|$$

where ${\Delta T}_{s}$ is the LST estimation error of the algorithms due to the error of the parameter x, Δx is the possible error of parameter x, x is the parameter. $T_{s}\left(x\right)$ and $T_{s}\left(x+\Delta x\right)$ represent the retrieved LST when the parameters are x and x+Δx, respectively.

To analyze the sensitivity of an algorithm to a parameter, other parameters should be assumed to be fixed. For example, to perform a sensitivity analysis on the effective mean atmospheric temperature of the MWA, it should be assumed that the LSE and atmospheric transmittance are known. Then the impact of the possible estimation error of the effective mean atmospheric temperature on the LST error should be investigated. Among these algorithms mentioned above, the MWA needs three parameters: the effective mean atmospheric temperature, the LSE, and atmospheric transmittance; the SWA needs two parameters: the LSE and atmospheric transmittance; the SC method needs two parameters: the LSE and the water vapor content. Considering that the atmospheric transmittance can be calculated from the water vapor content, when performing sensitivity analysis, the input parameters to be analyzed are the LSE, the water vapor content, and the effective mean atmospheric temperature.

#### 2.4.1. Sensitivity Analysis of the MWA

As mentioned above, the MWA requires a sensitivity analysis for three parameters: the LSE, the water vapor content, and the effective mean atmospheric temperature. In the study area, the atmospheric water vapor content ω was 2.09 g/cm^2^ when the satellite overpassed, and the near surface air temperature was approximately 302 K, so the effective mean atmospheric temperature can be assumed to be 296 K. For the LSE of band 10 of the study area, it was found that the value of the LSE was concentrated between 0.96 and 0.98, so we assumed that the LSE was 0.97 and the error range as ±0.01. The count of the brightness temperature value of band 10 and the brightness temperature value was found to be concentrated between 295 K and 315 K, so we analyzed the influence of the error of each parameter on the result in this interval.

##### Sensitivity Analysis to LSE

**Figure 5:**
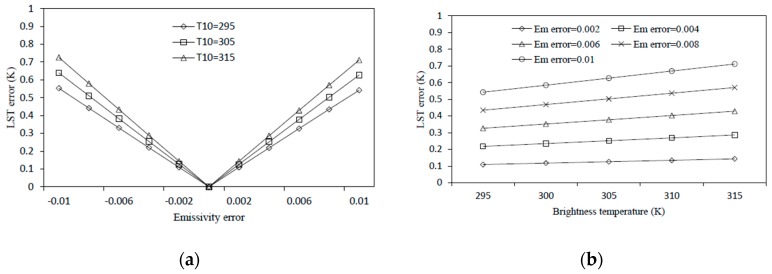
The LST (Land Surface Temperature) estimation error of mono-window algorithm as a result of the estimation error in LSE. (**a**) LST error against Emissivity error, and (**b**) LST error against brightness temperature for different emissivity error.

Figure 5a reflects how LSE estimation error influences the LST estimation error of the MWA under different brightness temperature conditions. Research has shown that the error in the estimation of LSE is usually ≤0.006 [10], so the surface emissivity error has a maximum impact of about 0.4 K. In addition, it can be seen from Figure 5a, that when the LSE error was constant, its influence on the result was different under different brightness temperatures. Figure 5b reflects when the estimation error of LSE was constant, the estimation error of the LST varied with the brightness temperature. It can be seen that, in the case where the surface radiance error was 0.006, as the brightness temperature increased from 295 K to 315 K, the error of the result increased by only 0.1 K. Therefore, the influence of the LSE error on the retrieved LST was less affected by the temperature changes.

##### Sensitivity Analysis to Water Vapor Content

**Figure 6:**
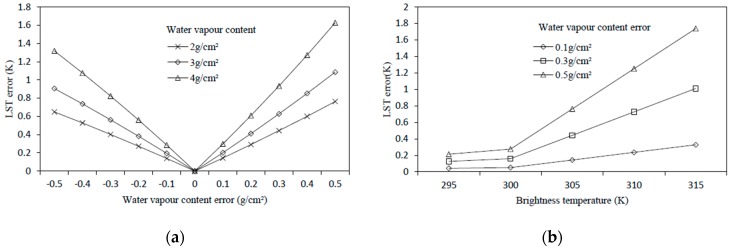
LST estimation error of the mono-window algorithm as a result of the estimation error in the water vapor content. (**a**) LST error against water vapor content error, and (**b**) LST error against brightness temperature for different water vapor content errors; water vapor content was kept constant at 2 g/cm^2^.

Liston et al. have indicated that the error of spatialized air humidity is usually smaller than ±5% [39], so it can be expected that the estimation error of water vapor content is in the range of ±0.3 g/cm^2^.Therefore, sensitivity analysis to water vapor content should be investigated separately when the atmospheric water vapor content is 2 g/cm^2^, 3 g/cm^2^, and 4 g/cm^2^ in the error range of ±0.5 g/cm^2^. From Figure 6a, it can be seen that at the water vapor content of 2 g/cm^2^, when the water vapor content error reaches 0.3 g/cm^2^, the result error reaches 0.4 K; and the higher the water vapor content, the more sensitive the algorithm is to the error of atmospheric water vapor content. It can be seen from Figure 6b that in the case of water vapor content error is constant. When the brightness temperature is higher than 295 K, the higher the brightness temperature, the greater the error of the result. Therefore, the MWA is sensitive to the error of water vapor content in a high temperature and humid environment.

##### Sensitivity Analysis to Effective Mean Atmosphere Temperature

Combining Formulas (4) and (25) gets Formula (26). It can be seen from Formula (26) that the influence of the error of the effective mean atmosphere temperature on the retrieved LST was related to the ratio of $D_{10}/C_{10}$, so it was necessary to examine the effect of the $T_{a}$’s error on the results in the case of different $D_{10}/C_{10}$. The ratio of $D_{10}/C_{10}$ was calculated at the LSE range of 0.96~0.99 and atmospheric transmittance of 0.7~0.9. The value of $D_{10}/C_{10}$ is shown in Table 7.

(26) $${\Delta T}_{s}=\left|\frac{D_{10}}{C_{10}}\times {\Delta T}_{a}\right|$$

**Figure 7:**
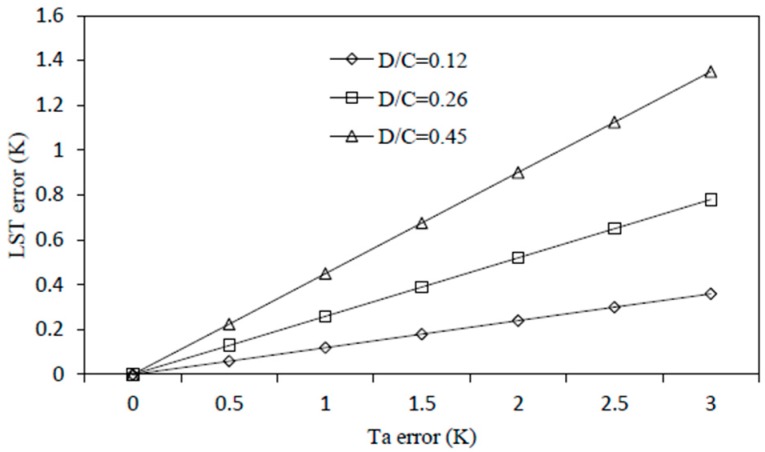
LST estimation error of mono-window algorithm as a result of the estimation error in near surface air temperature.

From the table, the values of $D_{10}/C_{10}$ can be assumed to be 0.12, 0.26, and 0.45. Since the difference in temperature data recorded by the weather station was within 2 K, so it can be expected that the error of the effective mean atmosphere temperature was less than 2 K. Therefore, sensitivity analyses should be performed when the $T_{a}$’s estimation error is within 3 K. It can be seen from Figure 7 that, when the error of $T_{a}$ reaches the maximum 2 K, the error of the result exceeded 0.8 K at the maximum.

According to the sensitivity analysis of the MWA, we found that the estimation error of atmospheric water vapor content had the greatest influence on the error of the retrieved LST, especially in the high temperature and humid environment, and the error of the result was more than 1 K. Secondly, the effective mean atmosphere temperature had the second largest impact on the retrieved LST; the error of the effective mean atmosphere temperature will cause the error of the retrieved LST to increase by approximately 0.8 K in extreme cases. The MWA had the lowest sensitivity to the LSE error. When the estimation error of LSE reached 0.006, the error of the result was only 0.4 K.

#### 2.4.2. Sensitivity Analysis of the SWA

The input parameters of the SWA are atmospheric transmittance and the LSE, and the atmospheric transmittance is a function of the water vapor content. Therefore, the sensitivity analysis of the SWA included the sensitivity analysis to the atmospheric water vapor content and the LSE. The SWA requires two thermal infrared bands, so there are two corresponding brightness temperatures. Therefore, the sensitivity analysis of the SWA needed to be performed under different $T_{10}$ and $T_{11}$ conditions. According to a study by Rozenstein et al. [14], the estimation error of the SWA is independent of the temperature change, and the statistical histogram of $T_{10}$–$T_{11}$ shows that the value of $T_{10}$–$T_{11}$ was between 0.5 K and 3.5 K, so we assumed the value of $T_{10}$ was 300 K and analyzed the sensitivity of the algorithm with $T_{10}$–$T_{11}$ in the range of 0.3–0.5 K.

##### Sensitivity Analysis to the Water Vapor Content

**Figure 8:**
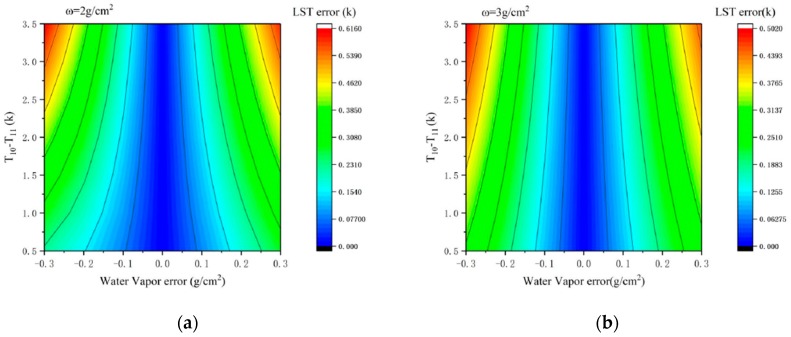
LST estimation error of the split window algorithm in the case of different $T_{10}$–$T_{11}$ as a result of the estimation error in water vapor content. (**a**) sensitivity of the SWA in the case of atmospheric water vapor content of 2 g/cm^2^, and (**b**) sensitivity of the algorithm in the case of atmospheric water vapor content of 3 g/cm^2^.

The sensitivity analysis of the SWA to atmospheric water vapor content is to analyze the influence of the estimation error of the water vapor on the LST retrieved by the SWA under different air humidity conditions. We discuss the sensitivity of the algorithm in the case of atmospheric water vapor content of 2 g/cm^2^ (Figure 8a) and 3 g/cm^2^ (Figure 8b), separately. Assuming $\varepsilon_{10}$ = 0.970 and $\varepsilon_{11}$ = 0.973, the error of the LST retrieved by the SWA is affected by the error of atmospheric water vapor content in the case of different $T_{10}$–$T_{11}$ as is shown in Figure 8. It can be seen from the figure that, when the estimation error of the water vapor content reached 0.3g/cm^2^, the error of the LST retrieved by SWA reached 0.5 K, and the influence of the water vapor content error on the result became smaller as the water vapor content increased.

**Figure 9:**
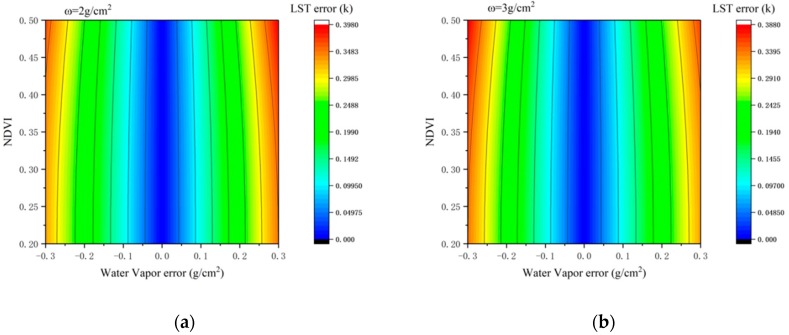
LST estimation error of the split window algorithm in the case of different LSEs as a result of the estimation error in water vapor content. (**a**) when the water vapor content is 2 g/cm^2^, (**b**) when the water vapor content is 3 g/cm^2^.

In order to further verify the sensitivity to atmospheric water vapor content, it was assumed that $T_{10}$–$T_{11}$ was a fixed value of 1.5 K. The error of the LST retrieved by the SWA was affected by the error of the atmospheric water vapor content in different pixels as is shown in Figure 9. It can be seen from the figure that, in the conditions of different air humidity, when the estimation error of the water vapor content reached 0.3 g/cm^2^, the error of the LST was approximately 0.4 K, and the magnitude of the error was not obvious with the change of the pixels.

##### Sensitivity Analysis to the LSE

**Figure 10:**
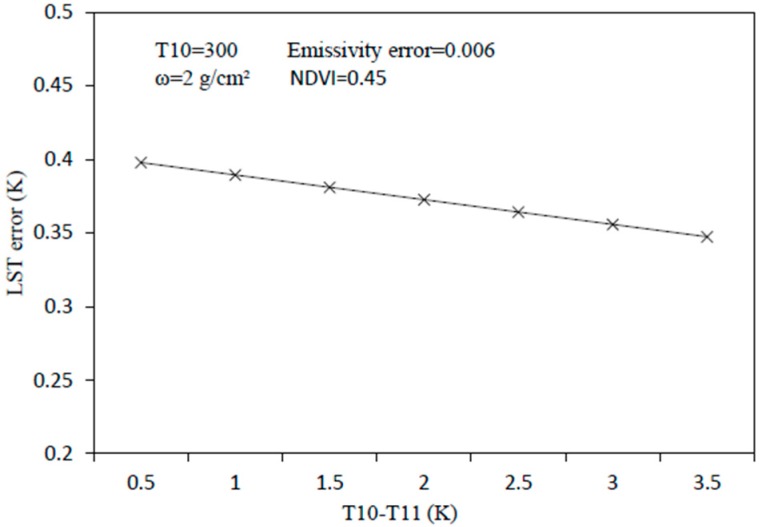
LST estimation error of the split window algorithm in the case of different $T_{10}$–$T_{11}$ as a result of the estimation error in LSE.

Estimation errors may occur simultaneously for $\varepsilon_{10}$ and $\varepsilon_{11}$, and it is also possible that the estimation errors of $\varepsilon_{10}$ and $\varepsilon_{11}$ are different. For convenience, we only considered the first kind of situation. Assume that the atmospheric water vapor content is 2 g/cm^2^, NDVI is 0.45, and estimation error of the LSE is 0.006. The variation of the LST error in the $T_{10}$–$T_{11}$ range of 0.5–3.5 is discussed. The results are shown in Figure 10. It can be seen from the figure that the error range of the LST was between 0.39 and 0.35. That is to say, the influence of different $T_{10}$–$T_{11}$ on the error of the LST was only 0.04 K, so the sensitivity analysis of the LSE did not need to consider different $T_{10}$–$T_{11}$ cases. Therefore, assuming that $T_{10}$–$T_{11}$ is a fixed value of 1.5, the sensitivity of the SWA to the LSE can be considered in this case.

**Figure 11:**
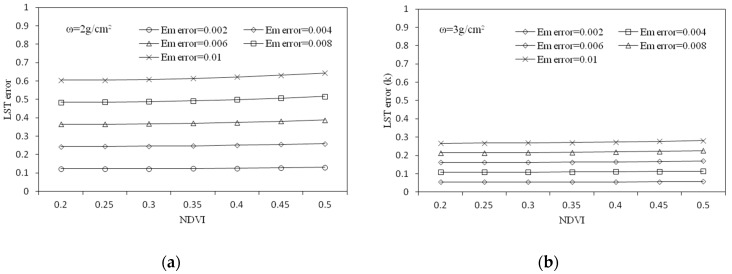
LST estimation error of the split window algorithm in the case of different LSEs as a result of the estimation error in LSE. (**a**) when the water vapor content is 2 g/cm^2^, (**b**) when the water vapor content is 3 g/cm^2^.

The following figures are the cases of $T_{10}$ − $T_{11}=1.5$K and the atmospheric water vapor contents were 2 g/cm^2^ (Figure 11a) and 3 g/cm^2^ (Figure 11b), separately, and the influences of the estimation error of LSE on the LST at different pixels. It can be seen from the figure that, when the error of the LSE was constant, the influence of the LSE error on the LST was almost the same in all the pixels. When ω = 2 g/cm^2^ and the LSE error was 0.006, the error of the LST was approximately 0.4 K. As the water vapor content increased, the sensitivity of the algorithm to the surface emissivity significantly reduced.

Through the sensitivity analysis of the SWA, we found that the maximum error of atmospheric water vapor content and LSE will increase the error of the LST by 0.4 K, and this effect will decrease as the water vapor content increases.

#### 2.4.3. Sensitivity Analysis of the SC Method

##### Sensitivity Analysis to Water Vapor Content

**Figure 12:**
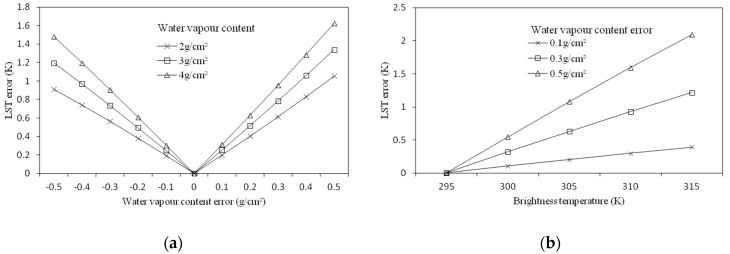
LST estimation error of the single-channel method as a result of the estimation error in the water vapor content. (**a**) LST error against water vapor content error, and (**b**) LST error against brightness temperature for different water vapor content error; water vapor content was kept constant at 2.09 g/cm^2^.

To analyze the sensitivity of the SC method to atmospheric water vapor content, firstly, the brightness temperature was assumed to be 305 K and then the influence of the error in the water vapor content on the LST at different humidity conditions was investigated. As can be seen from Figure 12a, under the circumstance of the atmospheric water vapor content at 2 g/cm^2^, when the estimation error of the water vapor content reached 0.3 g/cm^2^, the error of the LST was close to 0.6 K, and as the water vapor content increased, the influence of the water vapor content estimation error increased. We further investigated the influence of the estimation error of atmospheric water vapor content on the retrieved LST at different temperatures. We assumed that the atmospheric water vapor content was 2.09 g/cm^2^, then we investigated the influence of errors on the retrieved LST at different temperatures when the atmospheric water vapor content error was 0.1 g/cm^2^, 0.3 g/cm^2^, and 0.5 g/cm^2^. As shown in Figure 12b, when the water vapor content error reached 0.3 g/cm^2^, the error of the LST increased significantly with the increase in temperature. When the brightness temperature rose from 295 K to 315 K, the error of the retrieved LST increased by 1 K.

##### Sensitivity Analysis to LSE

**Figure 13:**
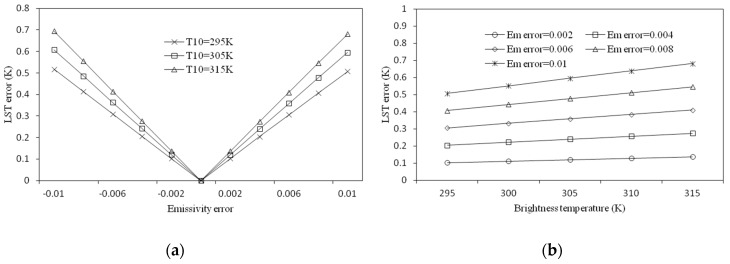
LST estimation error of the single-channel method as a result of the estimation error in LSE. (**a**) LST error against the emissivity error and (**b**) LST error against brightness temperature for different emissivity error.

The sensitivity analysis of the SC method to the LSE analyzes the influence of the estimation error of the LSE on the retrieved LST under different temperature conditions. We assumed ω = 2.09 g/cm^2^ and investigated the influence of the estimation error of the LSE to the retrieved LST, in the cases of $T_{10}$ = 295 K, $T_{10}$ = 305 K, and $T_{10}$ = 315 K, respectively. It can be seen from Figure 13a that, in the case of $T_{10}$ = 295 K, when the error of the LSE was 0.006, the error of the LST was approximately 0.3 K and the error increased as the temperature increased. In addition, the analysis of the influence of the LSE estimation error on the retrieved LST under different temperature conditions was performed. First, assuming that ω = 2.09 g/cm^2^, then as the temperature rose, we analyzed the influence of the error of the LSE on the LST in cases where the error of the LSE was 0.002, 0.004, 0.006, 0.008, and 0.01.

As shown in Figure 13b, when the error of the LSE was constant, its influence on the LST increased slowly as the temperature increased.

Through the sensitivity analysis of the SC method, we found that the error in atmospheric water vapor content will lead to larger errors in the LST, and this effect will increase with the increase in temperature and humidity, and in the case where the atmospheric water vapor content is 2.09 g/cm^2^, the estimation error of the water vapor content can lead to the maximum error of 1 K on the LST. In contrast, the error of the LSE had little effect on the error of the result, and the maximum influence was only 0.4 K.

## 3. Results

### 3.1. LST Retrieved by Three Methods

**Figure 14:**
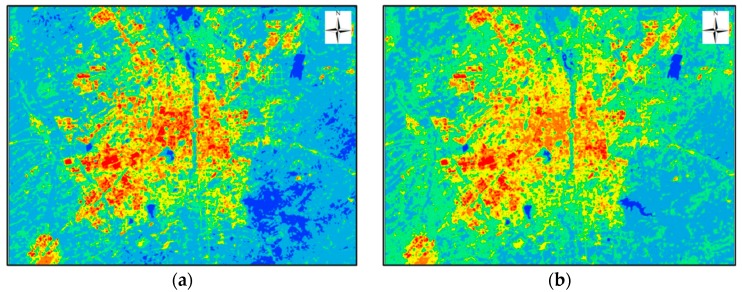

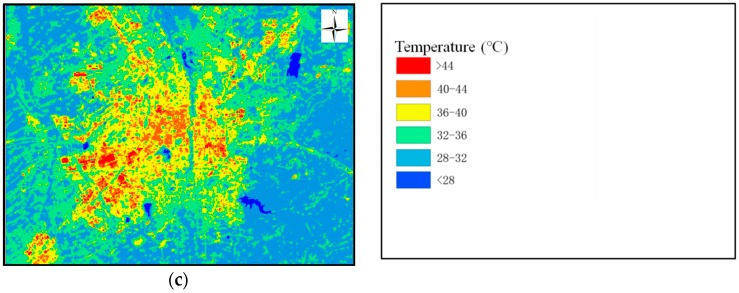
Land surface temperature in Changchun City where (**a**) is a result of the MWA, (**b**) is the result of the SWA, and (**c**) is the result of the SC method.

We used these three methods to retrieve the LST of Changchun City from Landsat 8 images, and the results are shown in Figure 14. It can be seen from the figure that the spatial variation of the LST retrieved by the three algorithms was roughly the same, and the surface temperature of the urban area was significantly higher than that of the suburbs. Three different algorithms yielded similar LST results which indicates that all three algorithms can retrieve LST well, and the results of the three methods can be used to study urban heat island effects, but the accuracy of these results needs further verification. 

### 3.2. Verification of the Retrieved LST

After retrieving the LST, the results needed to be verified to ensure the correctness of the results. There are three common methods for verifying the result: The first is a radiance-based method; this method uses atmospheric simulation tools, such as MODTRAN, to simulate the top radiance of the atmosphere by inputting parameters, such as LST, atmospheric parameters, and emissivity, and continuously changes the input LST until the simulated top radiance of the atmosphere is consistent with the atmospheric radiance observed by the sensor. Then, the input LST can be regarded as the true temperature of the land surface. Comparing this temperature with the retrieved one can verify the correctness of the results. Qin et al. [10] used this method when studying the LST of the Israeli–Egyptian border. The advantage of this method is that the LST can be accurately verified by computer simulation. The disadvantage is that atmospheric parameters and the LSE are difficult to obtain, and the simulation process is complicated. The second is to compare the retrieved LST with the MODIS LST product, called the cross-comparison method. Since the MODIS satellite overpass time is almost identical to the Landsat 8 overpass time—although the spatial resolution of the MODIS surface temperature product is low (1000 m)—and if the retrieved LST has the same spatial characteristics with the MODIS LST product, we can draw the conclusion that results are of good accuracy. Yang et al. [29] used this method when inverting the surface temperature of Shihezi. The advantage of this method is that MODIS surface temperature products are free and easy to obtain. The disadvantage is that MODIS surface temperature products data may loss in some areas, and because the spatial resolution is not high, the correctness of the retrieved results can only be roughly verified. The third method is to compare with ground observation data. This method is the simplest in theory—just by comparing the retrieved LST with the LST measured in the field, the accuracy of the retrieved LST can be verified [16]. The advantage of this method is that it is simple and accurate. The disadvantage is that it is tough and expensive to obtain verification data, and it is not feasible for studying past surface temperatures, because it is impossible to return to the time of satellite overpass to measure accurate surface temperature.

Since the data of MODIS LST products in this study area are missing, and the air temperature data of the local meteorological station was obtained in advance, we selected the third verification method, verifying the result by comparing the retrieved LST with the air temperature obtained by local meteorological stations. As described above, this method had the following problems: (1) The spatial resolution of the Landsat 8 TIRS data was 100 m. Whether the measured temperature can represent the temperature of this pixel is uncertain. (2) It is impossible to return to the time point of the satellite overpass time to measure the verification temperature, posing challenges if we are going to study the LST of a past period of time. (3) The verification of the LST requires many measured data; it is costly and time and labor consuming work to measure as much temperature data as possible to cover the study area at the same time. Therefore, this method should be improved.

It is known that in areas with high vegetation coverage, the vegetation canopy temperature is approximately equal to the air temperature [40,41,42]. Therefore, it is possible to select an area with a high vegetation coverage and use the air temperature recorded by the meteorological station to verify the retrieved LST. The air temperature data recorded by the meteorological station had good time continuity, and the weather stations were evenly distributed within the city. Therefore, it was a better way to verify the results using the air temperature recorded by the meteorological stations. However, it should be noted that, because there is no pure vegetation pixel (in the extent of one pixel, there was only vegetation) in the city, the air temperature will be slightly lower than the surface temperature, because the main source of air temperature is ground radiation conduction.

**Figure 15:**
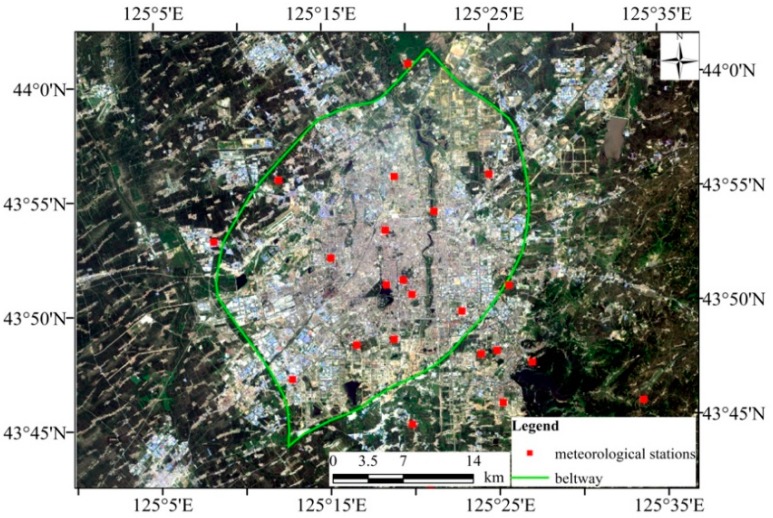
The distribution of the meteorological stations.

The distribution of Changchun City meteorological stations is shown in Figure 15. Ten of the 23 meteorological stations are located in areas with high vegetation coverage. The air temperature at the time of satellite overpass recorded by them was used to verify the LST of the three algorithms. The results are shown in Table 8. 

**Figure 16:**
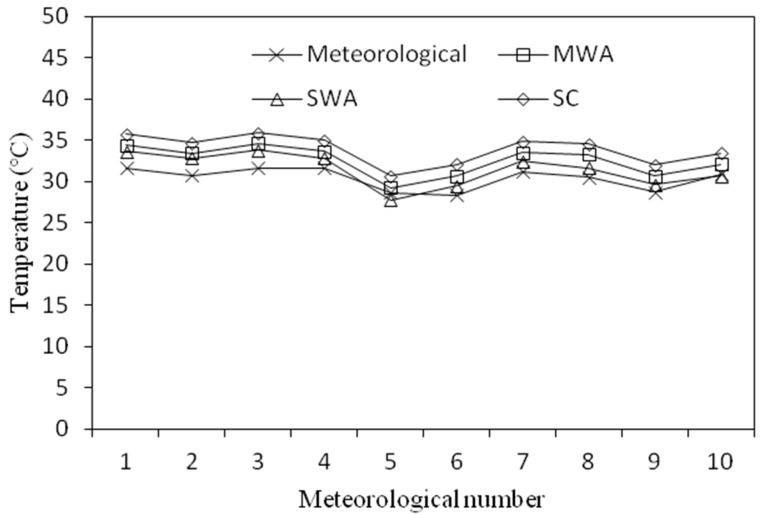
Comparison of the calculation results of the three algorithms with the local meteorological data.

It can be seen from the table that the average temperature difference between the LST calculated by the MWA and the air temperature measured by the meteorological station was 2.16 °C, and the RMSE was 0.72; the average temperature difference of the results of the SWA was 1.08 °C, and the RMSE was 0.94; The SC method had an average temperature difference of 3.5 °C and RMSE of 0.71. These results are reasonable and consistent with the results of Yang et al. [16,22,43], according to their study, the retrieved LST was about 6 K and 3 K higher than the air temperature for the summer and the winter dates, respectively. Thus, the results of these three methods are credible. From Figure 16, we see that the retrieved LST had the same curve as the air temperature. Therefore, we can conclude that all three methods can retrieve an LST that can reflect the spatial distribution of urban land surface temperature.

## 4. Discussion

Comparing the sensitivity analysis of the three algorithms, we found that the SWA had the lowest sensitivity to the error of input parameters, and errors in the parameters will have a smaller impact on the results in humid environments. The MWA and the SC method were more sensitive to errors in the input parameters, especially in hot and humid conditions. These two algorithms were more sensitive to errors in the atmospheric water vapor content. When the error of atmospheric water vapor content reached the maximum of 0.3 g/cm^2^, the error of the LST exceeded 1 K. Compared with the atmospheric water vapor content, both algorithms were less sensitive to the error of the LSE; when the estimation error of the LSE reached the maximum of 0.006, the error of the result reached 0.4 K. In addition, the MWA was also sensitive to errors in the effective mean atmosphere temperature. As far as the results of the sensitivity analysis are concerned, the SWA was the most stable method, and the error of the input parameters did not cause a large error in the LST. The second as the SC method; compared with the MWA, it did not require the effective mean atmosphere temperature to be an input parameter; the possibility of LST error was thus greatly reduced. The MWA had the highest sensitivity. In a humid and high temperature environment, same as the SC method, the MWA was very sensitive to errors in atmospheric water vapor content. But more input parameters greatly increased the possibility of an error in the result.

In the third part, Changchun City was taken as an example to retrieve the LST using the three methods. The results show that the spatial distribution of the urban thermal environment calculated by the three algorithms was basically the same. This means that all three algorithms can extract the LST accurately. In order to further verify the accuracy of the LST retrieved by three methods, based on the comparison of various verification methods, this paper proposed to use the air temperature data recorded by meteorological stations located in areas with high vegetation coverage to verify the retrieved LST. This method is based on the fact that in areas with high vegetation coverage, the LST is approximately equal to the air temperature. The results showed that all these three methods can retrieve the LST properly. Comparing these three methods, the MWA requires the most input parameters, namely, the LSE, the atmospheric transmittance, and the effective mean atmosphere temperature. Both the SWA and the SC method require only two parameters: the LSE and atmospheric transmittance. The difference is that the SWA requires two thermal infrared bands, so the corresponding LSE and atmospheric transmittance are both doubled. When extracting the LST from the Landsat 8 TIR data, it is necessary to select an appropriate method based on the condition of the study area and the obtained data. In high temperature and humid areas, the MWA and SC method are very sensitive to the error of atmospheric water vapor content. Therefore, the SWA is recommended. In the case of low temperature and water vapor content, all three algorithms can be used. Since the MWA is sensitive to the error of the effective mean atmosphere temperature, if a more accurate effective mean atmosphere temperature cannot be obtained, it is recommended to use the SWA and the SC method.

## Figures and Tables

**Table 1:** Constants for computing brightness temperature from Landsat 8 Thermal Infrared Sensor (TIR) data.

	M	A	$\;K_{1}$ $\left(W\cdot m^{-2}\cdot sr^{-1}\cdot \mu m^{-1}\right)$	$\;K_{2}\;(K)$
Band 10	0.0003342	0.1	774.89	1321.08
Band 11	0.0003342	0.1	480.89	1201.14

**Table 2:** Determination of coefficients a_10_ and b_10_ for the Landsat 8 TIR Band 10.

Temperature Range	a_10_	b_10_	$\;R^{2}$
20–70 °C	−70.1775	0.4581	0.9997
0–50 °C	−62.7182	0.4339	0.9996
−20–30 °C	−55.4276	0.4086	0.9996

**Table 3:** The emissivity values of water, vegetation, and soil for Landsat 8 TIRS band 10 and band 11.

	$\varepsilon_{\mathrm{water}}$	$\varepsilon_{\mathrm{soil}}$	$\varepsilon_{\mathrm{vegetation}}$	$\varepsilon_{\mathrm{Galvanized}-\mathrm{Steel}}$	$\varepsilon_{\mathrm{red}\;\mathrm{roof}}$
Band 10	0.991	0.984	0.964	0.959	0.958
Band 11	0.986	0.980	0.970	0.962	0.969

**Table 4:** Linear relations for the approximation of effective mean atmospheric temperature (T_a_) from the near surface air temperature ($T_{0}$).

Atmospheres	Linear Relations Equations
Tropical model	T_a_ = 17.9769 + 0.9172 $T_{0}$
Mid-latitude summer	T_a_ = 16.0110 + 0.9262 $T_{0}$
Mid-latitude winter	T_a_ = 19.2704 + 0.9112 $T_{0}$

**Table 5:** Relationship between atmospheric transmittance and water vapor content in the column for the water vapor content range of 0.5-3 g·cm^−2^.

Profile	Estimation Equation	*R* ^2^	SEE (Standard Error of Estimate)
1976 US Standard	$\tau_{10}$ = −0.1146ω + 1.0286	0.9882	0.0094
	$\tau_{11}$ = −0.1568ω + 1.0083	0.9947	0.0086
Mid-latitude summer	$\tau_{10}$ = −0.1134ω + 1.0335	0.986	0.0101
	$\tau_{11}$ = −0.1546ω + 1.0078	0.996	0.0073

**Table 6:** Determination of coefficients $a_{10},\;b_{10},\;a_{11}$ and $b_{11}$ for the Landsat 8 TIR Band 10 and Band 11.

T Range (°C)	$a_{10}$	$\;b_{10}$	$\;r_{10}^{2}$	$\mathrm{SE}E_{10}$	$\;a_{11}$	$\;b_{11}$	$\;r_{11}^{2}$	$\;\mathrm{SE}E_{11}$
0–30	−59.1391	0.4213	0.9991	0.0424	−63.3921	0.4565	0.9991	0.0438
0–40	−60.9196	0.4276	0.9985	0.0746	−65.2240	0.4629	0.9985	0.0769
10–40	−62.8065	0.4338	0.9992	0.0415	−67.1728	0.4694	0.9992	0.0427
10–50	−64.6081	0.4399	0.9986	0.0730	−69.0215	0.4756	0.9986	0.0750

**Table 7:** The ratio of $D_{10}/C_{10}$ in the case of different LSE (Land surface emissivity) and transmission.

Emissivity	Transmission	$C_{10}$	$D_{10}$	$D_{10}/C_{10}$	Average Ratio
0.96	0.7	0.672	0.3084	0.458929	0.447351
0.97	0.7	0.679	0.3063	0.451105	
0.98	0.7	0.686	0.3042	0.44344	
0.99	0.7	0.693	0.3021	0.435931	
0.96	0.8	0.768	0.2064	0.26875	0.261599
0.97	0.8	0.776	0.2048	0.263918	
0.98	0.8	0.784	0.2032	0.259184	
0.99	0.8	0.792	0.2016	0.254545	
0.96	0.9	0.864	0.1036	0.119907	0.116553
0.97	0.9	0.873	0.1027	0.11764	
0.98	0.9	0.882	0.1018	0.11542	
0.99	0.9	0.891	0.1009	0.113244	

**Table 8:** Comparison of the calculation results of the three algorithms with local meteorological data.

Number	Local Meteorological	Mono-Window Algorithm		Split Window Algorithm		Single Channel Method	
	Temperature (°C)	Temperature (°C)	Δ	Temperature (°C)	Δ	Temperature (°C)	Δ
1	31.60	34.38	2.78	33.63	2.03	35.72	4.12
2	30.70	33.40	2.70	32.79	2.09	34.71	4.01
3	31.60	34.59	2.99	33.78	2.18	35.90	4.30
4	31.65	33.63	1.98	32.83	1.18	34.97	3.32
5	28.65	29.26	0.61	27.76	−0.89	30.63	1.98
6	28.35	30.69	2.34	29.47	1.12	32.04	3.69
7	31.15	33.46	2.31	32.45	1.30	34.82	3.67
8	30.50	33.26	2.76	31.66	1.16	34.56	4.06
9	28.70	30.62	1.92	29.58	0.88	32.00	3.30
10	30.9	32.09	1.19	30.66	−0.24	33.43	2.53
Average difference			2.16		1.08		3.5
RMSE			0.72		0.94		0.71
